# *OsBRKq1*, Related Grain Size Mapping, and Identification of Grain Shape Based on QTL Mapping in Rice

**DOI:** 10.3390/ijms22052289

**Published:** 2021-02-25

**Authors:** Jae-Ryoung Park, Dany Resolus, Kyung-Min Kim

**Affiliations:** 1 Division of Plant Biosciences, School of Applied Biosciences, College of Agriculture and Life Science, Kyungpook National University, Daegu 41566, Korea; icd92@naver.com; 2 Coastal Agriculture Research Institute, Kyungpook National University, Daegu 41566, Korea; 3 Department of Crop Production, Faculté d’Agronomie et de Médecine Vétérinaire (FAMV), Université d’Etat D’Haiti (UEH), National Road number 1, Damien, Port-au-Prince 1441, Haiti; resolusdany@yahoo.fr

**Keywords:** rice, QTL, food shortage, yield, grain size, *OsBRKq1*

## Abstract

The world population is growing rapidly, and food shortage remains a critical issue. Quantitative trait locus (QTL) mapping is a statistical analytical method that uses both phenotypic and genotypic data. The purpose of QTL mapping is to determine the exact gene location for various complex traits. Increasing grain weight is a way to increase yield in rice. Genes related to grain size were mapped using the Samgang/Nagdong double haploid (SNDH) populations. Grain sizes were diversely distributed in SNDH 113 populations, and *OsBRKq1* was detected on chromosome 1 in an analysis of QTL mapping that used 1000 grain weight, grain length, and grain width. *OsBRKq1* exhibited high sequence similarity with the brassinosteroid leucine-rich repeat-receptor kinases of *Arabidopsis thaliana* and *Zea mays*. It was also predicted to have a similar function because of its high homology. *OsBRKq1* interacts with various grain-size control genes. Among the SNDH populations, the analysis of the relative expression level during the panicle formation stage of *OsBRKq1* in panicles of SNDH117, which has the largest grain size, and SNDH6, which has the smallest grain size, the relative expression level was significantly increased in SNDH117 panicles. SNDH populations have been advancing generations for 10 years; various genetic traits have been fixed and are currently being used as bridging parents. Therefore, the stable expression level of *OsBRKq1* was confirmed via QTL mapping. In the future, *OsBRKq1* can be effectively used to increase the yield of rice and solve food problems by increasing the size of seeds.

## 1. Introduction

Rice (*Oryza sativa* L.) is an important part of human food consumption worldwide [1,2]. In fact, rice is an essential crop that is consumed by more than half of the world’s population [3,4,5]. In Asia alone, more than two billion people obtain around 70% of their energy intake from rice and its derivatives [6]. The total area used to plant rice worldwide is estimated at 154 million hectares per year, and its production is around 600 million tons [7]. This production level is sufficient to feed the current population; however, over an extended period, this equilibrium may be reversed because of demographic explosion. The current rice production should be increased by at least 40% by 2030 to meet the growing demand of the population [8]. To achieve this, early-maturing and high-yield rice varieties that can adapt to agro-ecological conditions need to be developed. Therefore, it is necessary to combine traditional breeding techniques with modern biotechnology tools to meet the projected production demand.

This study is relevant because it advocates the use of quantitative trait loci (QTLs) related to rice yield and grain size, to develop new varieties of rice. The QTLs include genes that contribute to the formation and composition of quantitative traits [9,10]. QTL analysis is a statistical method that considers two types of information: phenotypic data (particularly trait measurements) and genotypic data (usually molecular markers) [11]. The purpose of QTL analysis is to explain the genetic basis of the variation in complex traits [12,13]. QTL analysis allows researchers in fields like agriculture to link complex phenotypes to specific regions of chromosomes [14]. Yield is a complex agronomic trait that is regulated by several genes. In the case of rice, it is characterized by three main components: the filled grain number per panicle, the number of panicles per plant, and the 1000 grain weight. In recent decades, significant progress has been made in the cloning of key QTLs that can control grain yield and its components. Complex agronomic traits from segregated population studies involving recombinant lines, F_2_ and progeny, crossover populations, and doubled haploids (DHs) have been reported since the 1980s [15]. A total of 2060 QTLs related to rice yield and its components had been identified up to March 2014 (http://www.gramene.org (accessed on 12 October 2020)). Although this progress has facilitated a better understanding of the regulatory mechanisms of rice production, the findings of QTL mapping and gene cloning did not significantly improve rice yield under field conditions, and the application of QTLs to the practice of selection was minimal [16]. This could be attributed to the dependence of quantitative traits on agro-ecological conditions. However, there is evidence that the 1000 grain weight is an agronomic trait that plays a predominant role in the adaptation of rice to agro-ecological conditions (i.e., sea-sons and cultivated areas), to achieve an ideal yield. Regulating the 1000 grain weight is one of the major objectives of rice breeding programs. The association between yield and its components and the grain characteristics should be investigated.

The food shortage problem is a serious debate around the world. Therefore, in this study, seed characteristics like grain length, grain width, and 1000 grain weight were investigated for QTL mapping related to grain size. Using these, QTL mapping related to grain size was performed. More specifically, it aimed to identify QTLs that could affect these traits and construct a framework linkage map to detect candidate genes associated with the variation in these agronomic traits. In additionm the QTL regions and open reading frames (ORFs) detected in this study can be effectively used to breed crops with large grain sizes.

## 2. Results

### 2.1. Phenotype Evaluation

**Figure 1:**
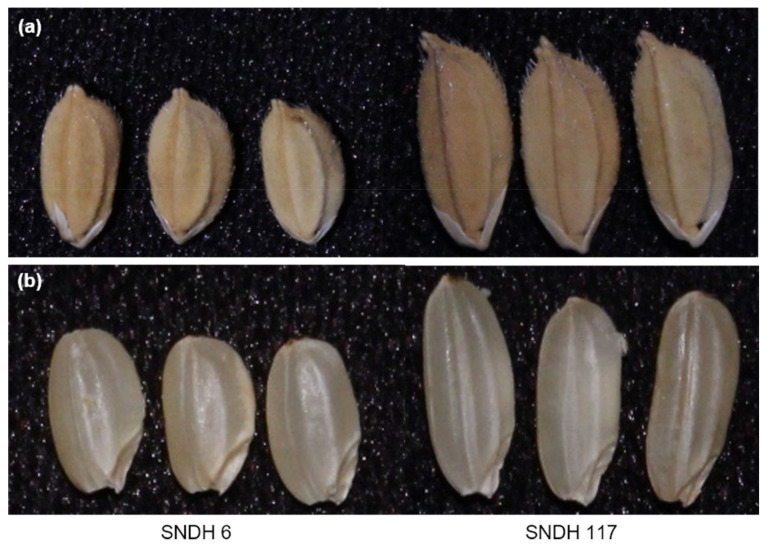
Mature paddy grains and brown rice gains of the SNDH 6 and SNDH 117 rice lines. Grain size varied in the SNDH (Samgang/Nagdong double haploid) line. The SNDH6 line had the smallest grain size, whereas SNDH117 had the largest grain size. When comparing the grain size before and after the chaff removal, the grain size of SNDH117 was the largest and the grain size of SNDH6 was the smallest. (**a**) Rough rice without removing the chaff. (**b**) Brown rice with chaff removed.

**Figure 2:**
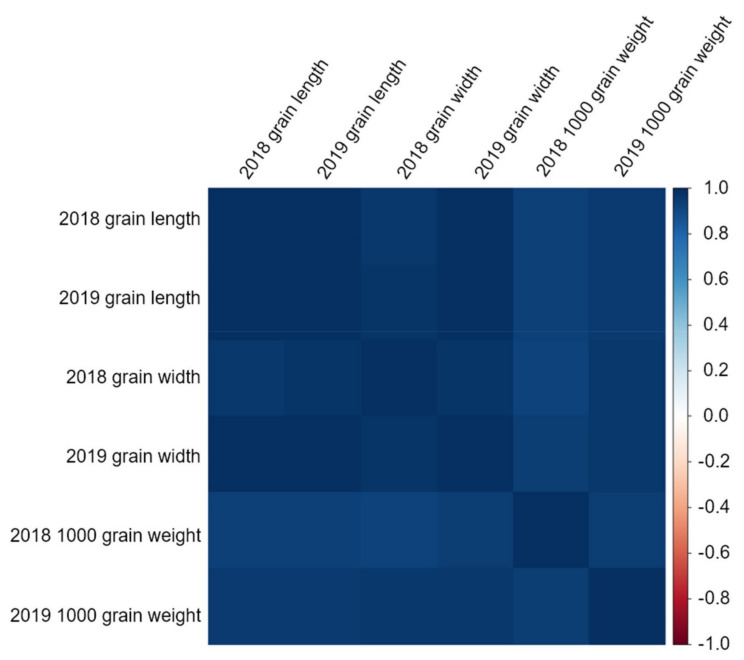
Correlation matrix heatmap with grain characteristics. The grain length, grain width, and 1000 grain weight all showed positive correlations. Therefore, grain shape is an important factor that can increase rice yield.

**Figure 3:**
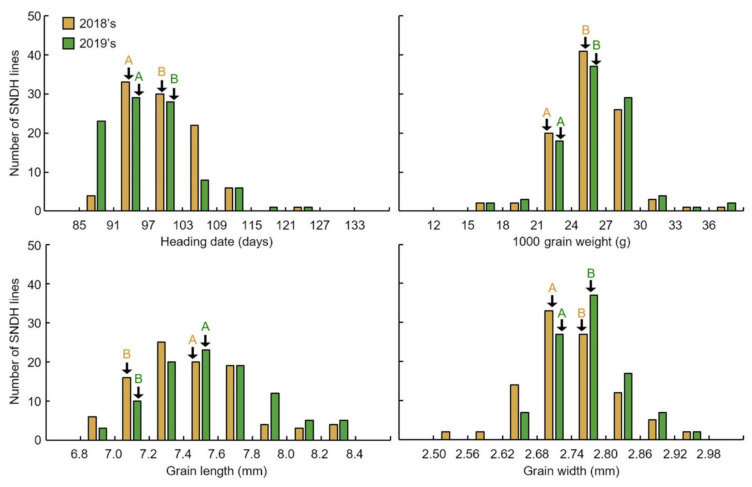
Frequency distribution for heading date, 1000 grain weight, grain length, and grain width in SNDH lines. Because all traits showed a normal distribution, the investigated traits can be considered quantitative traits. (**A**) Samgang; (**B**) Nagdong.

The association of between grain length, grain width, and 1000 grain weight was evaluated by QTL mapping. These data were obtained through 2 years of research (Figure 1 and Supplementary Table S1). In 2018, the grain length of the DH population (7.56 ± 0.34 mm) was closer to that of Samgang (7.60 ± 0.15 mm) than to that of Nagdong (7.36 ± 0.11 mm). Similarly, in 2019, the grain length of the DH population (7.53 ± 0.37 mm) was closer to that of Samgang (7.53 ± 0.12 mm) than to that of Nagdong (7.39 ± 0.13 mm). However, in 2018, the grain width of the DH population (2.77 ± 0.07 mm) was greater than that of the parents Nagdong (2.73 ± 0.02 mm) and Samgang (2.76 ± 0.03 mm). Similarly, in 2019, the grain width of the DH population (2.73 ± 0.05 mm) was greater than that of the parents Nagdong (2.75 ± 0.04 mm) and Samgang (2.71 ± 0.05 mm). In 2018, the 1000 grain weight of the DH population was moderate, with an average value of 22.89 g and a standard deviation of 3.10 g. The 1000 grain weight of Nagdong was 25.71 g with a standard deviation of 0.69 g, whereas that of Samgang was 21.57 g with a standard deviation of 0.38 g. In 2019, the 1000 grain weight of the DH population was moderate, with an average value of 23.58 g and a standard deviation of 2.15 g. The 1000 grain weight of Nagdong was 26.11 g with a standard deviation of 0.72 g, whereas that of Samgang was 22.01 g with a standard deviation of 0.27 g. Supplementary Table S2 presents the correlation between grain length, grain width, and 1000 grain weight. The three seed characteristics showed high correlations (Figure 2 and Supplementary Table S2). The frequency distributions of heading date, grain length, grain width, and 1000 grain weight in all Samgang/Nagdong double haploid (SNDH) populations showed continuous changes close to a normal distribution; therefore, they represented quantitative traits regulated by one or more genes (Figure 3). Because all traits were normally distributed, QTL mapping can be performed using these data.

### 2.2. QTL (Quantitative Trait Locus) Mapping for Grain Characteristics

**Figure 4:**
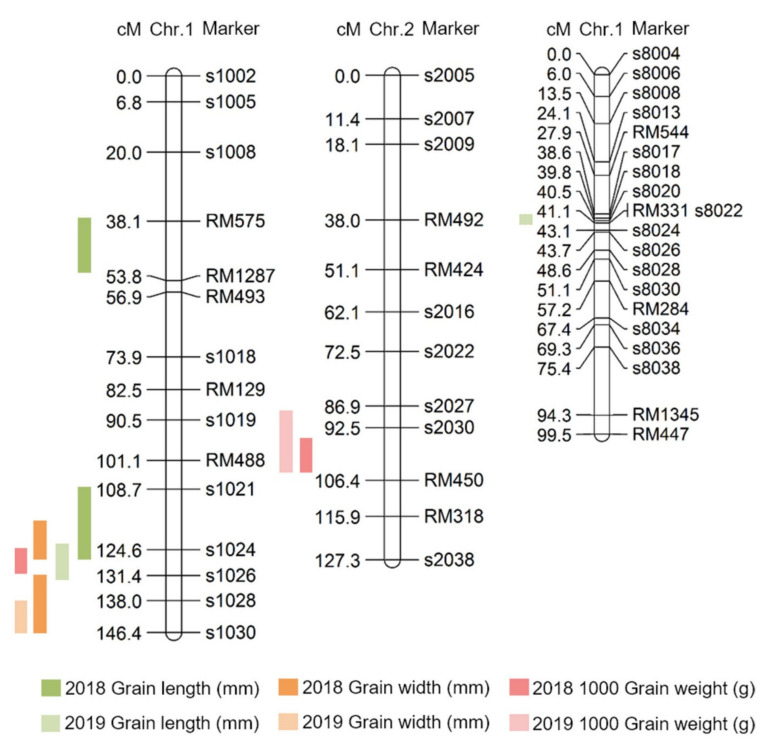
Chromosomal location of quantitative trait loci (QTLs) associated with grain size in the SNDH line. The QTLs mapped to chromosome 1, chromosome 2, and chromosome 8. Moreover, QTLs for all traits related to grain size were commonly located at s1024–s1026 on chromosome 1.

QTL (quantitative trait locus) mapping using the two-year phenotypic findings and analysis is presented in Supplementary Table S3 and Figure 4. A total of 850 SSR markers were used to construct the SNDH genetic map. Of these, 222 SSR markers with polymorphism in Samgang and Nagdong were finally selected and used to construct the SNDH genetic map. The total length of the genetic map constructed with 222 SSR markers was 2082.4 cM, and the interval between markers is 9.4 cM. These SSR markers are evenly distributed across 12 chromosomes. QTL mapping was performed using the phenotypic data collected in 2018 and 2019 to determine the QTL related to the grain length. Grain length, grain width, and 1000 grain weight were termed qGl, qGw, and qTgw, respectively. Two of these data were subjected to genetic mapping and QTL analysis for 2 years, and a total of 10 QTLs were obtained. In 2018, two QTLs (qGl1 and qGl1-1) were detected on chromosome 1, whereas in 2019, another two QTLs (qGl1-2 and qGl8) were identified on chromosomes 1 and 8, respectively. The qGl1 in 2018 was located at the RM575–RM1287 marker on chromosome 1, and yielded an LOD score of 2.8. The qGl1-1 (2018) was located at the s1021–s1024 marker on chromosome 1, and yielded an LOD score of 3.9. The qGl1-2 in 2019 was located at the s1024–s1026 marker on chromosome 1 and yielded an LOD score of 2.9. Lastly, the qGl8 (2019) was located at the s8017–s8018 marker on chromosome 8, and yielded an LOD score of 2.7. In addition, the QTL related to grain width (qGw), which can affect seed size, was found at a position similar to that detected for the grain length QTL. The qGw1 from 2018 was located at the s1026–s1030 marker on chromosome 1 and yielded an LOD score of 3.8. The qGw1-1 from 2019 was located at the s1021–s1024 marker on chromosome 1 and yielded a LOD score of 2.5. Meanwhile, the qGw1-2 (2019) was located at the s1028–s1030 marker on chromosome 1 and yielded an LOD score of 2.7. However, qGw1 (2018) originated from the allele of Samgang, and qGw 1-2 (2019) originated from the allele of Nagdong. Finally, the QTL region related to 1000 grain weight (qTgw) was searched. qTgw1 (2018) was located between s1024 and s1026 on chromosome 1 and yielded an LOD score of 2.4. The qTgw1-1 (2019) was located between s2030 and RM450 on chromosome 1 and yielded an LOD score of 3.3. The qGw2 (2019) was located at the s2027-RM450 marker on chromosome 2 and yielded an LOD score of 2.3. However, the qTgw in 2018 was derived from the allele of Nagdong, and the qTgw in 2019 was derived from the allele of Samgang (Supplementary Table S3). Over 2 years, one grain length, one grain width, and two 1000 grain weight QTLs were detected at approximately the same region.

### 2.3. Searching for Candidate Genes Related to Grain Characteristic

**Figure 5:**
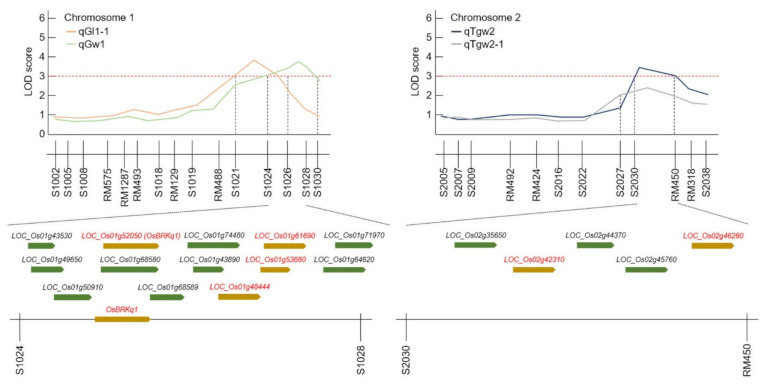
Quantitative trait locus (QTL) analysis and physical mapping related to grain-size candidate genes. After 2 years of QTL mapping, s1024–s1028 on chromosome 1 and s2030–RM450 on chromosome 2 were mapped consecutively. There were various candidate genes in this region, of which *OsBRKq1* on chromosome 1 was screened as a grain-size-associated gene.

The two-year, grain size-related chromosome 1 QTL mapping result was intensively searched for QTLs related to grain length, grain width, and 1000 grain weight. Among these regions, s1024–s1028 on chromosome 1 and s2030–RM450 on chromosome 2 were commonly detected regions related to grain size for the 2 years. Therefore, we searched for candidate genes related to grain shape, centering on s1024–s1028 on chromosome 1 and s2030–RM450 on chromosome 2. An analysis of simple sequence repeat (SSR) markers from NCBI in the s1024–s1028 region on chromosome 1 and the s2030–RM450 region on chromosome 2 revealed 18 ORFs (Figure 5). Among them, six ORFs (*LOC_Os01g52050*, *LOC_Os01g48444*, *LOC_Os01g53880*, *LOC_Os01g61690*, *LOC_Os02g42310*, and *LOC_Os02g46260*) were responsible for the functions related to grain shape (Supplementary Table S4). These ORFs corresponded to the brassinosteroid receptor kinase, auxin-responsive protein, and serine carboxypeptidase family protein.

### 2.4. Phylogenetic Tree and Homology Sequence Analyses

**Figure 6:**
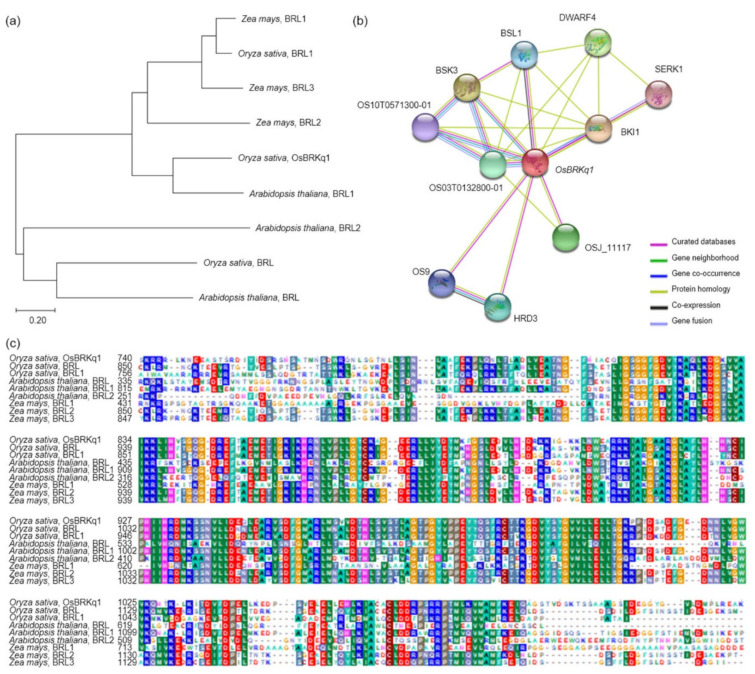
Sequence analysis of *OsBRKq1*. (**a**) Analysis of the relationships of the *OsBRKq1* gene and analysis of a homologous gene, using a phylogenetic tree that was constructed using 1000 bootstrap replicates. (**b**) Various proteins interacted with *OsBRKq1*. Brassinosteroids (BRs) regulate the growth and development all of plants. BRs regulate various pathways, such as the development of grain size, ethylene biosynthesis, and proton secretion into the cell wall. All of these proteins are related to the BR biosynthesis pathway. (**c**) Conserved domain of the protein sequences of homologous genes of *OsBRKq1*; very high similarity was observed in *Oryza sativa*, *Arabidopsis thaliana*, and *Zea mays*.

Among the candidate genes identified in the NCBI analysis, the sequence of *LOC_Os01g52050 (OsBRKq1)* was similar to that of the brassinosteroid LRR receptor kinase (BRL). Therefore, gene homology was compared and analyzed between *OsBRKq1* and brassinosteroid receptor kinases in *Arabidopsis thaliana*, *Oryza sativa*, and *Zea mays*. BLAST analysis showed that *OsBRKq1* had 68% sequence similarity with brassinosteroid insensitive 1-associated receptor kinase 1 of *Arabidopsis thaliana*. Phylogenetic tree analysis revealed that *OsBRKq1* had a very similar BRL sequence in *Zea mays*, in addition to BRL1 in *Arabidopsis thaliana*, and belonged to the same group (Figure 6a). Moreover, the results of the domain search for protein sequences showed very high similarity to *Arabidopsis thaliana*, *Oryza sativa*, and *Zea mays* (Figure 6c). Also, we used the OsBRKq1 domain to identify functional partners to predict the protein interactions of OsBRKq1. *OsBRKq1* interacted with 10 proteins (OS03T0132800-01, OS10T0571300-01, BSK3, BSL1, DWARF4, SERK1, BKI1, OSJ_11117, HRD3, and OS9) (Figure 6b).

### 2.5. Analysis of the Relative Expression Levels of Candidate Genes Related to Grain Size

**Figure 7:**
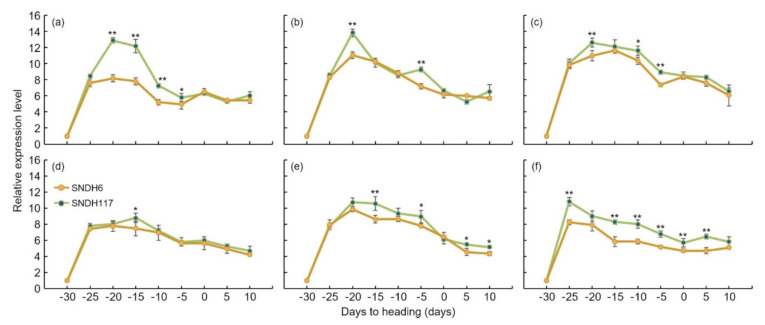
Analysis of the relative expression level of candidate genes related to grain size at the spikelet differentiation stage. Relative expression levels were analyzed at intervals of 5 days from 30 days before heading to 10 days after heading. The panicle formation stage starts from 30 days before heading, and grain size is determined at this time. The relative expression levels of candidate genes were analyzed in SNDH6 and SNDH117. * significant at the 0.05 level; ** significant at the 0.01 level. (**a**) *OsBRKq1*, (**b**) *LOC_Os01g48444*, (**c**) *LOC_Os01g53880*, (**d**) *LOC_Os01g61690*, (**e**) *LOC_Os02g42310*, and (**f**) *LOC_Os02g46260*.

**Figure 8:**
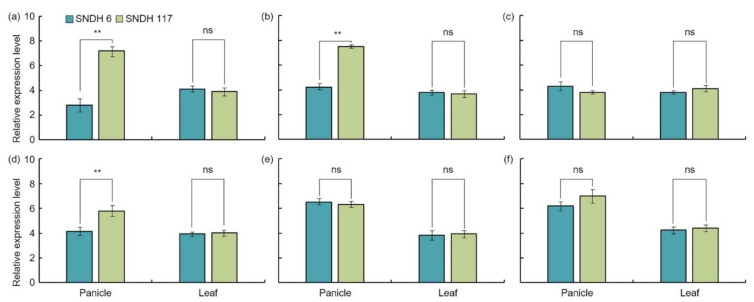
Comparison of relative gene expression levels in the leaves and panicles of the SNDH 6 and SNDH 117 lines, which had the smallest and largest grains, respectively, among the SNDH lines. There were no significant differences in any of the candidate genes in leaves. However, in the panicles, *LOC_Os01g48444* and *LOC_Os01g61690* were significantly different (*p* < 0.05) between the SNDH 6 and SNDH 117 lines. Moreover, *LOC_Os01g52050* was significantly different (*p* < 0.01) between the SNDH 6 and SNDH 117 lines in the panicles. ^ns^ no significant; ** significant at the 0.01 level. (**a**) *OsBRKq1*, (**b**) *LOC_Os01g48444*, (**c**) *LOC_Os01g53880*, (**d**) *LOC_Os01g61690*, (**e**) *LOC_Os02g42310*, and (**f**) *LOC_Os02g46260*.

Grain length, grain width, and 1000 grain weight related QTL mapping was performed and detected six candidate genes for grain size. The relative expression level of these candidate genes was checked in the SNDH6, which had the smallest grain size, and the SNDH117, which had the largest grain size. The relative expression levels of candidate genes related to grain size at the panicle formation stage were analyzed. *OsBRKq1* exhibited a significant difference in relative expression level in SNDH117 at the 1% level compared with SNDH6 on the 20th, 15th, and 10th days before heading. On the fifth day before heading, the relative expression level of *OsBRKq1* was high in SNDH117, with a significant difference at the 5% level. However, after heading, there was no significant difference in the expression of this gene between SNDH6 and SNDH117. *LOC_Os01g48444* exhibited a significant difference at the 1% level on the 20th and 5th days before heading, and its relative expression level was high in SNDH117. For *LOC_Os01g53880*, the relative expression level was high in SNDH117, with a significant difference at the 1% level on the 20th and 5th days before heading; in addition, the relative expression level detected in SNDH117 was high, with a significant difference at the 5% level on the tenth day before heading. *LOC_Os01g61690* had a high relative expression level in SNDH117 at the 5% level on the 15th day before heading. *LOC_Os02g42310* exhibited a significant difference at the 1% level on the 15th day before heading, and its relative expression level in SNDH117 was high. *LOC_Os02g46260* showed a high relative expression level in SNDH117, with a significant difference at the level of 1% at 25, 15, 10, and 5 days before heading; immediately after heading; and 5 days after heading (Figure 7). In addition, the comparison of the relative expression levels of leaf candidate genes revealed no significant differences among all candidate genes in SNDH6 and SNDH117 (Figure 8). However, the analysis of the relative expression levels of the candidate genes in panicles identified a significant difference between SNDH6 and SNDH117 (Figure 8). In particular, *OsBRKq1* showed a significant difference at the 1% level and a higher level of expression in SNDH117, which had a larger grain size. *LOC_Os01g48444*, *LOC_Os01g61690*, and *LOC_Os02g46260* showed significant differences at the 5% level between SNDH6 and SNDH117. In contrast, *LOC_Os01g53880* and *LOC_Os02g42310* showed no significant differences between SNDH6 and SNDH117.

## 3. Discussion

Grain length and grain width are related to 1000 grain weight. Here, the correlations between grain length, grain width, and 1000 grain weight were strongly positive (Supplementary Table S2). Moreover, as all values were close to 1, the grain length and grain width were closely related to the 1000 grain weight. Thus, studying the length and width of the grains can increase the 1000 grain weight, which leads to yield increase. Therefore, the traits investigated in this study were all related to 1000 grain weight and are important factors for increasing rice yield.

Here, QTLs for grain length, grain width, and 1000 grain weight were investigated using DH lines from a cross between Samgang and Nagdong, and were shown to be located on chromosome 1. To improve the accuracy of QTL mapping for grain length, grain width, and 1000 grain weight, 2 years of phenotypic data were examined, and the chromosomal location of QTLs was determined. A total of 10 QTLs were detected from the data collected in 2018 and 2019, and these QTLs were uniformly distributed on chromosomes 1, 2, and 8. The QTLs (qGl, qGw) related to the grain length and width, which determine the shape of the seeds, were all located at the s1028–s1030 marker on chromosome 1. The LOD scores for qGl (2018) and qGl (2019) were 3.9 and 2.9, respectively, while the LOD scores for qGw (2018) and qGw (2019) were 3.8 and 2.3, respectively. Wang et al. [17] conducted a QTL mapping of genes related to the grain size and shape of rice. They identified QTLs related to the grain shape at different locations on chromosome 1. This may be attributed to the different numbers of groups and markers used in that experiment. In this research, two-year data were used, and a high confidence level was observed because of the similar location and high LOD scores. The locations at which the LOD score exceeded 3.0 in at least one of the 2 years were mapped. All two-year data were mapped to the same marker position. Therefore, the gene that determines the shape of rice seeds was located at the s1028-s1030 marker on chromosome 1 of rice. qTgw1 (2018) and qTgw1 (2019) were located at the s1024–S1026 marker on chromosome 1, based on the QTL mapping of the 1000 grain weight. In addition, as the QTL position for the qTgw was close to the QTL position for the grain length and width, the weight of the seed may be affected by the shape of the seed.

The candidate genes identified in this study are all genes associated with increased seed size. A total of 18 candidate genes were detected in the QTL mapping, six of which were related to grain size. *OsBRKq1* exhibited sequence similarity with the brassinosteroid LRR receptor kinase. Brassinosteroids transcriptionally modulate seeds’ developmental pathways by binding to multiple seed development promoters, and affect seed size and shape through integuments, endosperm, and embryo development [18]. *LOC_Os01g48444* and *LOC_Os01g53880* exhibit sequence similarity with auxin-responsive protein IAA. Sun et al. [19] reported that the auxin response factor regulated a pathway upstream of auxin signaling, and was related to plant organ size and seed size. In addition, grain size increased when the auxin response factor was overexpressed [20]. *LOC_Os01g61690*, *LOC_Os02g42310*, and *LOC_Os02g46260* have serine carboxypeptidase-like sequences. Serine carboxypeptidase increases grain size by increasing cell proliferation and expansion in spikelet hulls [21]. In addition, serine carboxypeptidase affects the signaling of brassinosteroids and influences grain-size regulation [22]. The analysis of the relative expression levels of all of these candidate genes showed an absence of significant differences in the leaves. However, the analysis of the relative expression level of grain-size-related candidate genes in the panicles revealed a significant difference in *OsBRKq1* expression at the 1% level between the lines with the largest and smallest grain size. *LOC_Os01g48444*, *LOC_Os01g61690*, and *LOC_Os02g46260* also showed a significant difference in grain size at the 5% level between these two lines. Moreover, the size of the panicle was completely determined by the panicle formation stage. Therefore, in this study, the panicle formation stage was subdivided and sampled every 5 days, and the relative expression levels of all candidate genes were analyzed. *OsBRKq1* expression exhibited a significant difference in the initial stage of the spikelet differentiation stage, and its relative expression level was high in SNDH117. In SNDH6 and SNDH117, sequencing and alignment analyses were performed to determine whether the relative expression level of *OsBRKq1* was due to sequence variation; however, there was no difference in their sequences (data not shown). Grain size is considered the most important among the panicle formation stage [23]. Therefore, *OsBRKq1* detected in this study can be effectively used to control grain size. In particular, *OsBRKq1* is similar to systemin receptor SR160 precursor, and functions as a brassinosteroid LRR receptor kinase. Jiang et al. [24] explains that brassinosteroid genes are very important for seed control in *Arabidopsis*. In addition, not only brassinosteroids but also embryonic development plays an important role in seed size determination [25,26]. Dong et al. [27] also reported that embryonic development is important for seed size determination, and in that study, genes related to embryonic development were mapped onto chromosome 1, as in the present study.

The analysis of the interaction of *OsBRKq1* with intracellular proteins revealed that it interacts with a total of 10 proteins (OS03T0132800-01, OS10T0571300-01, BSK3, BSL1, DWARF4, SERK1, BKI1, OSJ_11117, HRD3, and OS9). First, *OsBRKq1* responds to brassinosteroids by binding to the brassinosteroid LRR receptor kinase, and regulates signals involved in plant development, cell kidney, and flowering promotion. *OsBRKq1* regulates grain size by interacting with BRI1, which is a negative regulator of brassinosteroid signal transduction. BSK3 is a serine/threonine protein kinase that acts as a positive regulator of the downstream brassinosteroid signal transduction of BRI1, and SERK1 acts as a positive regulator of somatic embryogenesis and the defense reaction of the LRR receptor kinase. DWARF4 catalyzes the hydroxylation step in brassinosteroid biosynthesis, while BSL1, OS10T0571300-01, and OS03T0132800-01 catalyze the reaction via protein phosphorylation. *OsBRKq1* interacts with OS9, HRD3, and OSJ_11117, which are proteins associated with quality control of the endoplasmic reticulum and endoplasmic-reticulum-associated degradation, in addition to the brassinosteroid biosynthetic reaction.

In this study, grain size-related QTL mapping was performed, which led to the search for various candidate genes at s1024–s1028 on chromosome 1 and s2030–RM450 on chromosome 2. Moreover, proteins with domains similar to those of these candidate genes have been reported previously as being associated with grain size. Ying et al. [28] mapped a major QTL for grain length and width on chromosome 3. Furthermore, Yosida et al. [29] mapped a rice grain-size-related QTL on chromosome 11. Thus, some studies have speculated that the differences in QTL mapping for the same trait may be due to differences in the materials used in the experiment, as well as genetic and environmental factors [30]. The present research identified the *OsBRKq1* gene, which was expressed in the spikelet differentiation stage and affected grain size. *OsBRKq1* is a putative causal gene, and can be effectively used to increase rice yield and investigate unknown grain size regulatory pathways. The results of this study may provide important information for increasing seed size and improving rice yield.

## 4. Materials and Methods

### 4.1. Rice Materials and Field Experiment

The rice Samgang/Nagdong double haploid (SNDH) line used here was the result of a cross between Samgang and Nagdong. This is a DH population composed of 113 lines. Samgang is an indica elite cultivar characterized by a heavy panicle and a high yield. In contrast, Nagdong is a japonica cultivar with good quality and many panicles per plant [31]. This study was conducted at the experimental field of Kyungpook National University in Gunwi. The planting density was 30 × 15 cm per plant. One row was transplanted for each line, and there were 23 plants in each row. Therefore, 23 plants were transplanted into one line. The N-P_2_O_5_-K_2_O level was 9.0/4.5/5.7 kg/10a; phosphoric acid and obscured fertilizer were used as the full base, and nitrogenous fertilizers were used with a base of 70% and a powder ratio of 30%. Herbicide and insecticide spraying was performed according to the standard rice cultivation method of the Rural Development Administration.

### 4.2. Phenotypic Evaluation

The 1000 grain weight associated with rice yield was investigated. In addition, the 1000 grain weight of the seeds was related to the shape of the seed; thus, the grain length and grain width of the seeds were measured together. Grains of the SNDH population 45 days after heading were harvested. After harvesting the SNDH line to investigate grain-size-related traits, ripening rice was carefully selected, and the grain length and width were measured using a caliper (caliper CD-15CP, Mitutoyo Corp., Kawasaki-shi, Kanagawa, Japan). To determine 1000 grain weight, 1000 grains of brown rice were repeatedly measured (five times), and the average value was used. The 1000 grain weight, seed length, and seed width were used for both the 2018 and 2019 examinations, for data reliability. The characteristics of grain size investigated in this research were repeatedly investigated (10 plants each) and statistically analyzed using the SPSS program (IBM SPSS Statistics, version 22, Armonk, NY, USA).

### 4.3. DNA Extraction

Leaf samples of the Samgang, Nagdong, and DH populations were collected and DNA was extracted. Healthy leaves (100 mg) of each line were placed in a 2 mL e-tube (MCT-200-C, AXYGEN, AZ, USA) with 3 mm beads, rapidly cooled with liquid nitrogen, and diluted with TissueLyser (TissueLyser II, QIAGEN, Hilden, Germany) for grinding. After adding 700 μL of DNA extraction buffer (2% CTAB (Cetyltrimethylammonium bromide), 0.1 M Tris pH 8.0, 1.4 M NaCl, and 1% PVP (Polyvinylpyrrolidone)) to the ground leaf sample, it was incubated for 20 min in a constant temperature bath at 65 °C. After the reaction, 700 μL of PCI (phenol/chloroform/isoamylalcohol = 25:24:1) was added. The sample was kept inverted at room temperature for 20 min, then centrifuged at 14,000 rpm for 10 min. After centrifugation, the supernatant was transferred to a 1.5 mL e-tube (microcentrifuge tubes, Sorenson, Murray, NY, USA). Subsequently, 350 μL of isopropanol was added to the separated supernatant, and the tube was inverted for 5 min and incubated at −72 °C for 2 h. After dissolving at room temperature, the sample was centrifuged at 13,000 rpm for 10 min, the supernatant was discarded, and the pellet was washed with 70% ethanol and dried. After drying, the DNA was dissolved in 20 μL of ddH_2_O, and the DNA concentration of each sample was adjusted to 20 ng/μL using a NanoDrop 2000 Spectrophotometer (ND-2000; Nanodrop, Waltham, MA, USA) for QTL analysis.

### 4.4. SNDH Gene Mapping

A total of 222 SSR markers were obtained from the Rural Development Administration and used to map the QTLs related to the heading date, seed length and width, and 1000 grain weight. PCR was performed in a total volume of 12 μL, which included 20 ng/μL genomic DNA, 200 μM dNTP (deoxynucleoside triphosphate)s, 0.1 U of Taq polymerase (RR001A, TaKaRa, Seoul, Korea), and 10 pmol primer. PCR (C1000, BioRad, Hercules, CA, USA) amplification was performed using pre-denaturation at 95 °C for 5 min, followed by 35 cycles of denaturation at 95 °C for 30 s, annealing at 55 °C for 30 s, and extension at 72 °C for 1 min; and a final extension at 72 °C for 7 min. The PCR products were separated using a vertical electrophoresis apparatus (NA-1114; NIHON EIDO Co. Ltd, Tokyo, Japan) at 350 V for 1 h and 30 min after loading in an 8% natural acrylamide gel. The gel was then stained with EtBr (Ethidium bromide), to confirm polymorphism.

### 4.5. QTL Analysis

Win QTL Cart 2.5 version was used for QTL analysis in this study. The effective use of this program requires multiple factors, such as the genome distance between markers, the label of the markers, the number of chromosomes, the genotypic data, and the number of target traits. After inserting all the data required by Win QTL Cart version 2.5, composite interval mapping at an LOD threshold of 2.5 was performed considering the entire genome [32,33].

### 4.6. Candidate Gene Information Analysis

The QTL analysis approximated the locations of the genes involved in 1000 grain weight, grain length, and grain width. Once the gaps between the markers were known, numerous genes in these regions were identified on the Rapdb (https://rapdb.dna.affrc.go.jp/ (accessed on 12 September 2020)) and Rice X pro (http://ricexpro.dna.affrc.go.jp/ (accessed on 15 September 2020)) websites. Candidate genes were selected from a number of genes obtained through these sites. Moreover, all sequences located between the two flanking markers at both ends of the QTL interval were downloaded, which corresponded to the QTL range. Each open reading frame (ORF) was sorted and analyzed according to function, and the shape associated with the target was filtered out. The Simple Modular Architecture Research Tool (http://smart.embl-heidelberg.de/ (accessed on 15 September 2020)) was used to predict the protein interactions of the selected candidate genes. In addition, NCBI (http://www.ncbi.nlm.nih.gov (accessed on 18 September 2020)) and BioEdit 7.0 (https://bioedit.software.informer.com/7.0/ (accessed on 18 September 2020)) were used for homology sequence analysis and gene sequence analysis.

### 4.7. Analysis of Relative Expression Levels Related to Candidate Genes for Grain Size

To check the relative expression levels of candidate genes, sampling was performed at the spikelet differentiation stage. The spikelet differentiation stage started at 30 days before heading, and sampling was performed every five days up to 10 days after heading. RNA was extracted from a sampled spikelet. Total RNA was extracted using the RNeasy plant mini kit (QIAGEN, Hilden, Germany) from the leaves and panicles of the SNDH6 (with the smallest grain size) and SNDH117 (with the largest grain size) lines, and 1 μg of RNA was used as a template for cDNA synthesis using transcriptase (Invitrogen, Carlsbad, CA, USA) and an oligodt primer. A qRCRBIO cDNA Synthesis kit (cat no. PB30.11-10, PCRBIOSYSTEM, Wayne, PA, USA) was used for cDNA synthesis. Quantitative real-time PCR was performed using an Eco Real-Time PCR system (Illmina, San Diego, CA, USA). *OsActin* was used as a control, each reaction was run in triplicate, and the mean and standard deviation were calculated.

### 4.8. Statistical Analysis

All heading date-, 1000 grain weight-, grain width-, and grain length-determining experiments in the SNDH lines were replicated at least five times each year, and all data were analyzed using the SPSS program (IBM SPSS Statistics, version22, Armonk, NY, USA).

## 5. Conclusions

Grain size is a very important factor for increasing rice yield. In this research, QTL mapping for grain length, grain width, and 1000 grain weight (which is related to grain size) was performed over a period of 2 years. The frequency distribution table for each trait revealed that all traits showed a normal distribution; moreover, it was found that they were quantitative traits, which implies continuous variation. Also, these three characteristics—grain length, grain width, and 1000 grain weight—are highly positively correlated. As a result of QTL mapping for 2 years, s1024–s1028 on chromosome 1 and s2030–RM450 on chromosome 2 were mapped at the same region. Six grain-size-related candidate genes (one brassinosteroid LRR receptor kinase, two auxin-responsive proteins, and three serine carboxypeptidases) were detected in the s1024–s1028 and s2030–RM450 regions. Among the SNDH lines, the relative expression levels of candidate genes in SNDH117, which has the largest grain size, and SNDH 6, which has the smallest grain size, were confirmed in leaves and panicles. In the leaves, none of the candidate genes showed significant differences. However, in the panicles, *OsBRKq1*, *LOC_Os01g48444*, and *LOC_Os01g61690* exhibited significant differences between SNDH 6 and SNDH 117. In particular, *OsBRKq1* showed a significant difference at the level of 1% between SNDH 6 and SNDH 117. This was the largest difference in relative expression level among candidate genes related to grain size; therefore, we examined *OsBRKq1* and found that it interacts with 10 proteins (OS03T0132800-01, OS10T0571300-01, BSK3, BSL1, DWARF4, SERK1, BKI1, OSJ_11117, HRD3, and OS9). All of these proteins are related to cell lodge and somatic embryogenesis. In addition, the homology analysis of *OsBRKq1* revealed that it had a very similar domain to the brassinosteroid LRR receptor kinase (BRL) of *Arabidopsis thaliana*, *Oryza sativa*, and *Zea mays*. Therefore, it is predicted that *OsBRKq1* will exhibit a similar function to that of the BRL proteins. *OsBRKq1*, which was newly discovered through this research, can be effectively used for the breeding of rice varieties with improved yield via an increase in grain size.

## Data Availability

The data presented in this study are available on request from the corresponding author.

## Supplementary Materials

The following are available online at https://www.mdpi.com/1422-0067/22/5/2289/s1, Table S1: The grain length, grain width and 1000-grain weight from the 113 SNDH lines, Table S2: Analysis of correlation between grain length, grain width, and 1000-grain weight, Table S3: QTL related to the grain length, grain width, and 1000-grain weight in the Samgang/Nagdong DH population, Table S4: Candidate genes related to grain length, grain width, and 1000 grain weight.
