# Supplementary material for: OsBRKq1, Related Grain Size Mapping, and Identification of Grain Shape Based on QTL Mapping in Rice
Source: Int J Mol Sci. 2021 Feb 25;22(5):2289. doi: 10.3390/ijms22052289 (PMC7956666; doi:10.3390/ijms22052289)
Supplement: Supplementary file 1 [file ijms-22-02289-s001.pdf]

Supplementary Materials

Supplementary Table S1. The grain length, grain width and 1000-grain weight from the 113 SNDH lines.

Plant trait	Years	Parents		DH population
		Samgang	Nagdong	
Grain length (mm)	2018	7.60 ± 0.15	7.36 ± 0.11	7.56 ± 0.34
	2019	7.53 ± 0.12	7.39 ± 0.13	7.53 ± 0.37
Grain width (mm)	2018	2.76 ± 0.03	2.71 ± 0.02	2.77 ± 0.07
	2019	2.71 ± 0.05	2.75 ± 0.04	2.73 ± 0.05
1000-grain weight (g)	2018	21.57 ± 0.38	25.71 ± 0.69	22.89 ± 3.10
	2019	22.01 ± 0.27	26.11 ± 0.72	23.58 ± 2.15

[a] The data are presented in mean ± standard deviation.

Supplementary Table S2. Analysis of correlation between grain length, grain width, and 1000-grain weight.

Plant trait	Grain length (mm)	Grain width (mm)	1000-grain weight (g)
Grain length (mm)	1		
Grain width (mm)	0.962**	1	
1000-grain weight (g)	0.930**	0.928**	1

** Correlation is significant at the 0.01 level. Population is 113 SNDH lines.

Supplementary Table S3. QTL related to the grain length, grain width, and 1000-grain weight in the Samgang/Nagdong DH population.

Chracteristics	Year	QTLs	Chr.	Interval Markers[z]	LOD	Additive effect[y]	r^{2x}	Increasing effects[w]
Grain length	2018	qGl1	1	RM575-RM1287	2.8	-0.5	0.2	Nagdong
(mm)		qGl1-1	1	s1021-s1024	3.9	1.2	0.1	Samgang
	2019	qGl1-2	1	s1024-s1026	2.9	-0.5	0.2	Nagdong
		qGl8	8	s8017-s8018	2.7	-0.9	0.1	Nagdong
Grain width	2018	qGw1	1	s1026-s1030	3.8	3.7	0.3	Samgang
(mm)		qGw1-1	1	s1021-s1024	2.5	0.4	0.1	Samgang
	2019	qGw1-2	1	s1028-s1030	2.7	-0.2	0.2	Nagdong
1000-grain weight	2018	qTgw1	1	s1024-s1026	2.4	-0.2	0.2	Nagdong
(g)		qTgw2	2	s2030-RM450	3.3	0.3	0.2	Samgang
	2019	qTgw2-1	2	s2027-RM450	2.3	-3.5	0.1	Nagdong

GL grain length, GW grain width and TGW thousand grain weight

[z] Interval markers are those within the significance threshold on each border of the QTL range

[y] The proportion of evaluated phenotype variation attributable to a particular QTL was estimated by the coefficient of determination (r^2)

[x] Positive values of the additive effect indicate that alleles from Samgang are in the direction of increasing the traits

[w] Increase allele is the source of the allele causing an increase in the measured trait.

Supplementary Table S4. Candidate genes related to grain length, grain width, and 1000 grain weight.

Chr.	Marker interval	Locus	Description
1	s1024-s1028	LOC_Os01g52050	Similar to Systemin receptor SR160 precursor (EC 2.7.1.37) (Brassinosteroid LRR receptor kinase)
		LOC_Os01g48444	Similar to Auxin-responsive protein IAA14 (Indoleacetic acid-induced protein 14)
		LOC_Os01g53880	Similar to Auxin-responsive protein IAA26 (Indoleacetic acid-induced protein 26)
		LOC_Os01g61690	Similar to Serine carboxypeptidase II-1 precursor (EC 3.4.16.6) (CP-MII.1) (Fragment)
2	s2030-RM450	LOC_Os02g42310	Similar to Serine carboxypeptidase II-like protein
		LOC_Os02g46260	Peptidase S10, serine carboxypeptidase family protein
